# Biologic therapies for severe pediatric asthma: efficacy, safety, and biomarker-guided selection

**DOI:** 10.3389/falgy.2026.1757445

**Published:** 2026-01-20

**Authors:** Enrico Tondina, Alessia Claudia Codazzi, Riccardo Castorina, Rossana Di Micco, Cristina Dutto, Livia Leoncini Bartoli, Giovanni Lepore, Gian Luigi Marseglia, Ilaria Brambilla

**Affiliations:** 1 Department of Clinical-Surgical, Diagnostic and Pediatric Sciences, University of Pavia, Pavia, Italy; 2 Pediatric Clinic, Fondazione IRCCS Policlinico San Matteo, Pavia, Italy

**Keywords:** benralizumab, biologic therapy, children, dupilumab, mepolizumab, omalizumab, severe asthma, tezepelumab

## Abstract

**Background:**

Severe pediatric asthma is a heterogeneous, high-burden disease marked by variable corticosteroid responsiveness, frequent exacerbations, and substantial impairment in quality of life. Advances in airway immunobiology, particularly the delineation of type-2 (T2) pathways (IgE, IL-5, IL-4/IL-13) and epithelial alarmins, have enabled the development of targeted biologic therapies for biomarker-defined patient subgroups.

**Objective:**

To synthesize current evidence on the efficacy and safety of biologic therapies for severe pediatric asthma and to translate biomarker-driven selection into practical clinical guidance, while outlining emerging therapeutic directions.

**Summary of findings:**

Targeted biologics, anti-IgE (omalizumab), anti-IL-5/IL-5Rα (mepolizumab, benralizumab; pediatric data for reslizumab remain limited), anti-IL-4Rα (dupilumab), and anti-TSLP (tezepelumab) improve disease control, reduce severe exacerbations, and enable steroid-sparing in appropriately selected children. Benefits are greatest in T2-high profiles, particularly with elevated blood eosinophils and/or fractional exhaled nitric oxide (FeNO), while tezepelumab shows efficacy across biomarker strata. Lung-function gains are modest to moderate but clinically meaningful. Persisting gaps include optimal treatment duration, stopping rules, long-term safety, cost, and equitable access.

**Conclusions:**

Biologic therapies have reshaped the care of severe pediatric asthma, operationalizing precision medicine through immunologic endotyping and biomarker-guided selection. Priorities now include standardized definitions of response and remission, robust long-term safety data, and strategies to ensure equitable access across diverse pediatric populations.

## 1 Introduction

Asthma is a chronic inflammatory disorder of the lower respiratory tract characterized by variable airflow limitation and respiratory symptoms, with substantial effects on quality of life and socioeconomic burden (1). Pediatric asthma remains highly prevalent globally, and environmental pressures, including tobacco smoke exposure, urbanization, and deteriorating air quality, continue to shape disease trajectories (2).

Approximately 2%–5% of treated children meet criteria for severe asthma, a small but high-impact subgroup disproportionately driving exacerbations, health-care use, and corticosteroid exposure (3).

According to international guidelines, including those of the European Respiratory Society (ERS) and the American Thoracic Society (ATS), severe asthma is defined as disease that remains uncontrolled despite optimized high-dose inhaled corticosteroids (ICS) plus at least one additional controller—or that worsens when this treatment is stepped down—after addressing modifiable factors (4).

Within this spectrum, difficult-to-treat asthma reflects poor control largely attributable to comorbidities, environmental exposures, suboptimal adherence, or inhaler misuse, whereas severe therapy-resistant asthma (STRA) remains uncontrolled despite systematic identification and correction of these contributors. Precise distinction prevents both unnecessary escalation and undertreatment (5).

Severe pediatric asthma is associated with high morbidity and disproportionate healthcare use, including recurrent emergency visits, hospital admissions, and prolonged pharmacotherapy, with substantial socioeconomic costs (6). Despite optimized treatment, many children continue to experience persistent symptoms, frequent exacerbations, and reduced lung function. Reliance on rescue and maintenance corticosteroids carries important risks—short-term increases in gastrointestinal bleeding, sepsis, and pneumonia, and long-term complications such as adrenal suppression, growth impairment, osteoporosis, diabetes, and recurrent respiratory infections (7, 8).

Although mortality is uncommon, children with severe asthma remain highly susceptible to acute exacerbations and to progressive, and in part irreversible, airway remodeling—changes that may track into adulthood and increase the risk of chronic obstructive pulmonary disease (COPD). Taken together, these risks underscore the need for early, comprehensive, and sustained management to optimize long-term outcomes (6).

Advances in asthma immunopathophysiology have enabled targeted biologic therapies for severe, persistent disease. Monoclonal antibodies directed against IgE (omalizumab), IL-5/IL-5Rα (mepolizumab, benralizumab, and reslizumab), and IL-4Rα (dupilumab) consistently reduce severe exacerbations, provide steroid-sparing benefits, and yield clinically meaningful improvements in lung function in appropriately selected children (9). In parallel, emerging evidence positions the airway epithelium as a proximal driver of inflammation via alarmins (e.g., TSLP). Targeting this axis with tezepelumab improves symptom control and reduces exacerbations, including in patients with lower T2 biomarker levels, although further studies are needed to clarify mechanisms, long-term safety, and durability of effect in pediatric populations (10).

Biologic selection is anchored to asthma phenotype/endotype after systematically addressing modifiable contributors to poor control. Decision-making is increasingly biomarker-guided, incorporating blood eosinophil count, fractional exhaled nitric oxide (FeNO), patterns of allergic sensitization, and total/allergen-specific IgE (11). In selected children, biologics have substantially improved disease control and reduced exacerbations, with meaningful steroid-sparing effects. Nevertheless, further work is needed to refine age-specific efficacy estimates, establish long-term safety and stopping rules, and validate novel or composite biomarkers that can better personalize treatment selection across pediatric subgroups.

This narrative review synthesizes current evidence on biologic therapies for severe pediatric asthma, covering mechanisms of action, clinical utility, efficacy and safety, and biomarker-guided criteria for patient selection, while highlighting knowledge gaps relevant to everyday practice.

We searched PubMed/MEDLINE (January 2000–July 2025) for English-language studies using combinations of: severe asthma, therapy-resistant, difficult-to-treat, child/pediatric, phenotype, endotype, biologic/targeted therapy, and specific agents (omalizumab, mepolizumab, benralizumab, reslizumab, dupilumab, tezepelumab). We prioritized randomized trials, meta-analyses, high-quality observational studies, and international guidance (e.g., ERS/ATS, GINA). Reference lists of recent reviews were screened for additional studies. Pediatric data were emphasized; adult evidence is cited only to contextualize mechanisms or when pediatric data were limited.

## 2 Pathophysiology of severe asthma

Asthma is a heterogeneous chronic inflammatory disease of the airways. Multiple exposures and traits, including aeroallergens, viral infections, obesity, hormonal influences, tobacco smoke, exercise and cold air, as well as genetic variants (e.g., ADAM33, PHF11, DPP10, GPRA), converge to sustain airway inflammation, ultimately leading to variable airflow limitation, hyperresponsiveness, and structural remodeling (12).

Immune dysregulation involves both innate and adaptive arms and is broadly classified into type-2 (T2)–high and non-T2 inflammatory pathways (13).

In children, T2-high disease predominates. Following allergen exposure, naïve CD4^+^ T cells polarize toward Th2 cells that secrete IL-4, IL-5, IL-9, and IL-13. IL-4 and IL-13 drive B-cell class switching to IgE; subsequent IgE cross-linking on mast cells triggers degranulation with histamine and leukotriene release, producing acute bronchoconstriction. IL-5 promotes eosinophil maturation, recruitment, and survival, whereas IL-9 and IL-13 contribute to mucus hypersecretion and airway hyperreactivity (12).

The airway epithelium functions as an upstream orchestrator of inflammation. Barrier dysfunction facilitates penetration of allergens and pathogens and induces the release of alarmins, notably TSLP, IL-25, and IL-33, which activate dendritic cells and group-2 innate lymphoid cells, amplify Th2 polarization, and sustain eosinophilic inflammation (10). This mechanistic framework underpins current biologic targets (IgE, IL-5/IL-5Rα, IL-4Rα) and supports upstream intervention at the epithelial–alarmin axis via TSLP.

IL-25 and IL-33 further amplify type-2 inflammation by activating group-2 innate lymphoid cells (ILC2s) and potentiating mast cells, eosinophils, and basophils, thereby enhancing downstream IL-5/IL-13 signaling, mucus hypersecretion, and airway hyperresponsiveness (14). T2-high asthma endotypes arise from the interplay of these epithelial-alarmin, innate, and adaptive immune pathways, which constitute the principal targets of current and emerging biologic therapies. Accumulating evidence has clarified these mechanisms, enabling more precise, biomarker-guided therapeutic strategies.

Although less prevalent than T2-high disease, non–T2 asthma endotypes occur in both adults and children and are increasingly recognized as clinically meaningful. Non-T2 asthma is driven predominantly by Th1/Th17 pathways, with release of pro-inflammatory cytokines such as IL-17, IL-6, and tumor necrosis factor-α (TNF-α). This pattern is frequently accompanied by neutrophilic or paucigranulocytic airway inflammation, relative corticosteroid unresponsiveness, and a more challenging clinical course. The underlying mechanisms remain incompletely defined, underscoring the need for further research to inform novel therapeutic strategies (15).

Advances in asthma immunology now support endotype-based classification and the use of personalized, biomarker-driven therapies to enhance disease control. Biomarkers are central to differentiating endotypes and directing targeted treatment. In clinical practice, the most widely applied markers are blood eosinophil count, fractional exhaled nitric oxide (FeNO), and serum total IgE. Importantly, T2 status is dynamic: approximately 30%–50% of patients shift across conventional T2 cutoffs over time, underscoring the need for repeated measurements and clinical context when making treatment decisions (16).

A blood eosinophil count ≥150 cells/µL indicates type-2 inflammation and correlates with exacerbation risk. Alone, or more strongly in combination with elevated FeNO (≥20 ppb in children), it signals high airway inflammation, poorer asthma control, greater likelihood of severe exacerbations (17). However, most evidence linking type-2 inflammatory biomarkers to asthma outcomes has been derived from adult populations, and pediatric-specific prognostic thresholds remain less well defined (18–20). Composite use of eosinophils and FeNO improves predictive accuracy compared with either marker alone and can help prioritize anti-IL-5/IL-5Rα or anti-IL-4Rα therapies. In pediatric asthma, the most direct evidence supporting biomarker-guided treatment selection comes from *post hoc* analyses of the LIBERTY ASTHMA VOYAGE trial, showing that baseline blood eosinophils and FeNO identify children with the greatest clinical benefit from dupilumab, including reductions in exacerbations and improvements in lung function (21).

Serum total IgE remains essential for omalizumab dosing, although its value for predicting response is limited. Longitudinal assessment of free IgE and related indices has been explored as a potential tool for treatment monitoring and pharmacodynamic assessment; however, these approaches remain investigational and are not currently validated for routine clinical use (22). By contrast, allergen-specific IgE reflects sensitization burden and has beeen associated with asthma severity and exacerbation risk. Current evidence does not support allergen-specific IgE as a reliable predictor of clinical response to omalizumab, particularly in pediatric populations, and it does not inform dose selection (23).

A range of emerging biomarkers, including periostin, serum TSLP, urinary eicosanoids (e.g., LTE₄), and selected microRNAs, is being evaluated to refine pediatric endotyping and to improve prediction of biologic response; however, most lack standardized assays, pediatric reference ranges, and prospective validation. More recently, nasal transcriptomic profiling has emerged as a promising, minimally invasive approach to capture airway inflammatory signatures and may represent a future tool to improve asthma endotyping and treatment stratification in pediatric populations, although its clinical applicability remains under investigation (24). Systemic IL-6 has been proposed as a marker of non–T2 phenotypes, but its clinical utility is constrained by poor disease specificity and susceptibility to confounding from concurrent inflammatory states (e.g., obesity, infection) (25, 26).

## 3 Biologic therapies approved in pediatric severe asthma

### 3.1 Anti-IgE (omalizumab)

Omalizumab is a recombinant, humanized monoclonal antibody that binds the C*ε*3 domain of free IgE, preventing IgE from engaging the high-affinity Fc*ε*RI on mast cells, basophils, and dendritic cells. By lowering circulating free IgE, omalizumab downregulates Fc*ε*RI expression on effector cells, blunts allergen-driven mediator release, and attenuates downstream type-2 inflammation and airway remodeling (27, 28).

Omalizumab was the first biologic authorized for asthma. It is approved by the EMA (2009) and the FDA (2016 for ages 6–11 years) for add-on treatment in patients ≥6 years with moderate-to-severe allergic asthma who have a positive skin test or *in-vitro* sensitization to a perennial aeroallergen and inadequate control despite high-dose ICS plus additional controller(s) (29). In the NICE pathway, omalizumab is recommended as an add-on in patients ≥6 years with severe allergic asthma, positive SPT or serum specific IgE, total IgE 30–1,500 IU/mL, and recurrent oral corticosteroid bursts (≥4 in the prior year) (30). GINA lists omalizumab as a Step 5 option for uncontrolled moderate-to-severe allergic asthma with recent exacerbations and perennial sensitization (31). Beyond asthma, omalizumab is indicated for chronic spontaneous urticaria and chronic rhinosinusitis with nasal polyps, and since 2024 it is also approved to reduce allergic reactions to multiple foods after accidental exposure in selected adults and in children ≥1 year (32).

For severe allergic asthma, omalizumab is administered subcutaneously every 2–4 weeks, with the dose determined by baseline total serum IgE and body weight; administration can occur in clinic or at home according to local practice and labeling (33).

Given its long clinical history in pediatric allergic asthma, omalizumab has been extensively evaluated across randomized trials, meta-analyses, and real-world studies. A Cochrane review of 25 RCTs (through 2013), including trials in children (5–12 years) and adolescents (12–17 years), showed a significant reduction in exacerbations requiring oral corticosteroids with omalizumab vs. placebo (odds ratio: 0.55, 95% CI: 0.42–0.60). Hospitalizations were also reduced (OR: 0.16, 95% CI: 0.06–0.42), with an absolute risk around 0.5% on omalizumab vs. 3% on placebo over 28–60 weeks. Patients receiving omalizumab were more likely to discontinue ICS (OR: 2.50, 95% CI: 2.00–3.13) and, when continued, reduced their daily ICS dose (weighted mean difference −118 μg/day beclomethasone dipropionate equivalent, 95% CI: −154 to −84). Effects on maintenance oral corticosteroid tapering were not statistically significant (OR: 1.18, 95% CI: 0.53–2.63) (34).

Pediatric RCTs corroborate these findings. In a double-blind trial of 334 children aged 6–12 years, 28 weeks of anti-IgE therapy enabled complete ICS withdrawal in many participants and reduced acute events (18.2% vs. 38.5%; *p* < 0.001) (35). The IA-05 study (627 children) reported a 43% reduction in the annualized exacerbation rate over 52 weeks vs. placebo, rising to 50% among those on high-dose ICS plus LABA (36). In the 60-week ICATA trial (419 inner-city youth, 60% aged 6–11 years), omalizumab reduced asthma symptom days by 24.5%, exacerbations, and use of ICS/LABA; *post-hoc* analyses suggested attenuation of the seasonal (viral-associated) exacerbation peaks (37). Mechanistically, omalizumab has been shown to enhance plasmacytoid dendritic cell IFN-α responses and downregulate Fc*ε*RIα expression, effects that may contribute to the observed clinical benefit (38).

Findings from real-world cohorts corroborate RCT evidence. In a French multicenter study of 104 children (aged 6–18 years), 12 months of omalizumab was associated with a 72% reduction in severe exacerbations, an 88.5% reduction in hospitalizations, a 30% decrease in daily ICS dose, and improved FEV_1_; >80% of patients maintained these gains at 2 years (39). In an Italian multicenter cohort of 47 children (57% <12 years), exacerbations fell by 91%, hospitalizations by 96%, maintenance OCS were discontinued in 100%, and FEV_1_ increased from 79% to 91% predicted (40).

Collectively, these data support the durable efficacy and favorable safety of omalizumab in routine pediatric practice (41).

Biomarker data indicate that omalizumab is most effective in children with T2-high profiles, particularly those with elevated FeNO and blood eosinophil counts (BEC) (42). In the EXTRA analysis, reductions in annualized exacerbation rate (AER) were greatest in participants with FeNO ≥19.5 ppb (AER change: −53%, 95% CI: −70 to −37; *p* < 0.001) and in those with BEC ≥260 cells/μL (AER change: −32%, 95% CI: −48 to −11; *p* = 0.005) (43). By contrast, baseline total or allergen-specific IgE shows limited predictive value for clinical response. Recent work has explored longitudinal free IgE and baseline periostin as adjunct markers to refine response prediction, but these approaches require prospective validation (23, 44). Although eligibility and dosing for omalizumab are determined by total serum IgE (with weight-based algorithms), total IgE should not be interpreted as a standalone predictor of treatment benefit; integrating FeNO and eosinophil counts provides more actionable guidance for patient selection (42).

Regarding lung function, omalizumab has been associated with FEV_1_ improvements particularly in adolescents, with gains most evident in patients exhibiting high T2 biomarker profiles (e.g., FeNO ≥25 ppb and BEC ≥300 cells/μL); effects are not universal across all treated children (45). Additional features linked to better response include polysensitization and higher total IgE, whereas weaker responses have been reported with older age (>12 years), recent exacerbations, lower baseline FEV_1_ (<90% predicted), and comorbidities such as obesity, gastroesophageal reflux, nasal polyposis, and psychological conditions (46, 47).

The PARK randomized trial is testing whether early anti-IgE intervention in high-risk toddlers (aged 2–3 years) can prevent or modify subsequent asthma by blunting IgE-mediated responses over a 4-year course (48). In the context of allergen immunotherapy (AIT), adjunctive omalizumab has been shown to shorten time to maintenance dosing and reduce adverse events, potentially improving AIT tolerability; however, this use is off-label and requires further controlled pediatric data (49). Across pediatric studies, omalizumab has demonstrated a favorable safety and tolerability profile (50). The most common adverse events are injection-site reactions (reported in up to ∼45%, typically mild and transient) (51). Other frequently reported events include headache, pharyngitis, and upper respiratory tract infections (approximately 5%–10%). Serious adverse events are uncommon; reports include appendicitis, pneumonia, and bronchitis, with a low estimated risk of anaphylaxis (52).

The optimal duration of omalizumab in children with severe allergic asthma remains unsettled, and there is no consensus on safe withdrawal criteria (41). Nevertheless, real-world data suggest that discontinuation may be feasible in carefully selected patients who achieve sustained control (41).

In a large French database analysis, Humbert et al. reported maintenance of asthma control in 76%, 44%, and 33% of pediatric patients at 1, 2, and 3 years after stopping therapy, respectively—outcomes more favorable than those observed in adults (53).

An Italian retrospective series (Foti et al.) documented persisting clinical benefits up to 12 months post-discontinuation, including improved lung function, fewer exacerbations, reduced OCS use, and better quality of life (54). In a prospective cohort of 20 children, Ferraro et al. found that withdrawal appeared safe but emphasized the need for close monitoring given the limited evidence base (55).

Taken together, these findings indicate that omalizumab cessation can be considered in eligible pediatric patients with stable control, provided that monitoring is structured and prompt re-initiation is available; however, prospective studies are required to define selection criteria, timing, and post-withdrawal follow-up protocols (41).

### 3.2 Anti–IL-5 and anti–IL 5 R (mepolizumab—benralizumab—reslizumab—depemokimab)

The IL-5 axis is central to eosinophilic airway inflammation. Mepolizumab and reslizumab are monoclonal antibodies that neutralize IL-5, thereby reducing eosinophil activation, survival, and trafficking. Benralizumab binds IL-5Rα on eosinophils and basophils and triggers antibody-dependent cellular cytotoxicity (ADCC), producing rapid and near-complete eosinophil depletion (56).

According to GINA, mepolizumab is approved as a Step 5 add-on for severe eosinophilic asthma in children ≥6 years, typically characterized by blood eosinophils ≥150 cells/µL and recurrent exacerbations despite high-dose ICS plus at least one additional controller (31).

Recommended dosing is 40 mg SC every 4 weeks for ages 6–11 years and 100 mg SC every 4 weeks for ≥12 years, administered via pre-filled syringes (57).

Pediatric studies show robust pharmacodynamic and clinical effects. In an open-label PK/PD study of children 6–11 years, 12 weeks of mepolizumab reduced blood eosinophils by ∼88.5% (40 mg) and ∼83.4% (100 mg), with post-treatment means of 42 and 55 cells/µL, respectively—consistent with reductions seen in adolescents and adults—supporting biological efficacy in the pediatric population (58).

The MUPPITS-2 (Mechanisms Underlying Asthma Exacerbations Prevented and Persistent With Immune-Based Therapy: A Systems Approach Phase 2) randomized, placebo-controlled trial enrolled 290 urban children aged 6–17 years and provided robust evidence for mepolizumab's efficacy. Treatment reduced the annualized exacerbation rate by 27% vs. placebo (rate ratio: 0.73; *p* = 0.027). Transcriptomic profiling of nasal samples showed selective downregulation of eosinophil-associated gene modules—including type-2 pathways and eicosanoid metabolism—that were linked to exacerbation risk in the placebo group but were attenuated with mepolizumab. In contrast, epithelial signaling (e.g., IL-33 responses and EGFR pathways) remained active or became upregulated among children with persistent symptoms, suggesting that non-eosinophilic inflammatory circuits can sustain disease activity despite IL-5 blockade and may inform adjacent or combination therapeutic strategies (59).

Findings from Fricker et al. and Vultaggio et al. suggest that mepolizumab preferentially depletes inflammatory eosinophils—characterized by low CD62L—while sparing subsets involved in tissue homeostasis. This selective depletion supports an immune rebalancing model rather than wholesale eosinophil eradication, implying that functional phenotype, not just eosinophil number, influences treatment response (60, 61).

The SCOUT substudy deepened this concept of eosinophil heterogeneity using high-dimensional CyTOF on induced sputum from MUPPITS-2 participants. Three eosinophil populations were identified by CD62L expression (low, intermediate, high). Children on mepolizumab who nevertheless experienced exacerbations had higher proportions of CD62L-intermediate/high eosinophils, which showed upregulated activation markers (CD69, CCR3) and increased cytokine secretion (IL-5, IL-13, TNF-α). These data indicate that, despite peripheral eosinophil suppression, activated airway subsets can persist and may drive breakthrough exacerbations (62).

Extended follow-up supports a favorable safety profile for mepolizumab in children with severe eosinophilic asthma. In an open-label extension of patients aged 6–17 years, serious adverse events were uncommon and none were attributed to treatment. The most frequent events were mild infections and headache; only a single event was considered drug-related. Notably, no cases of anaphylaxis or systemic hypersensitivity were reported. Although pediatric numbers were modest, these findings are consistent with earlier trials and support the continued use of mepolizumab in this population (63).

A recent secondary analysis of the MUPPITS-2 trial profiled respiratory illnesses in urban children with eosinophilic, exacerbation-prone asthma randomized to mepolizumab or placebo. Among mepolizumab-treated participants, breakthrough exacerbations were driven predominantly by upregulation of non-T2 inflammatory pathways, whereas persistent T2 activity accounted for only a minority of episodes. Suppression of eosinophil-mediated T2 inflammation appeared to unmask alternative immunopathologic circuits—including epithelial activation, tissue-remodeling programs, macrophage-associated responses, mucus hypersecretion, and cellular stress—that contributed to residual disease activity. Distinct molecular exacerbation endotypes were also identified according to the presence or absence of concomitant viral infection. Collectively, these findings refine our understanding of mepolizumab's transcriptomic effects and point to adjacent therapeutic targets for pediatric patients with exacerbation-prone asthma (64). In summary, mepolizumab is effective for many children with severe eosinophilic asthma; however, airway eosinophil heterogeneity and non–T2 pathways can limit response, reinforcing a biomarker-guided approach and raising the possibility of rational combination strategies in selected cases.

Importantly, despite the established role of blood eosinophils as eligibility criteria for anti–IL-5 therapy, analyses specifically evaluating type-2 inflammatory biomarkers as predictors of clinical response to mepolizumab in pediatric populations are currently lacking. This represents a relevant evidence gap and limits the direct extrapolation of biomarker-guided treatment algorithms derived from adult studies to children.

Among anti–IL-5 therapies, benralizumab targets IL-5Rα on eosinophils and basophils, triggering antibody-dependent cellular cytotoxicity (ADCC) and producing rapid, near-complete, and sustained eosinophil depletion (57).

In the UK, benralizumab is recommended for adults with uncontrolled severe eosinophilic asthma (SEA) who meet any of the following: BEC ≥0.3 × 10^9^/L with ≥4 OCS-treated exacerbations in the prior 12 months; ongoing maintenance OCS ≥5 mg prednisolone/day within the past 6 months; or ≥3 exacerbations with BEC ≥0.4 × 10^9^/L in the past year (65).

Regulatory approvals have expanded from adults (FDA 2017) to adolescents ≥12 years (2019) and most recently to children 6–11 years. Pediatric dosing is weight-based: 30 mg SC for children ≥35 kg and 10 mg SC for those <35 kg. The regimen includes an induction phase (three doses at 4-week intervals) followed by maintenance every 8 weeks (66).

The pivotal phase 3 SIROCCO and CALIMA trials that established the efficacy of benralizumab enrolled patients aged 12–75 years. Although adolescents were included, prespecified subgroup analyses did not demonstrate clear or consistent efficacy trends in participants younger than 18 years. Therefore, the evidence base supporting benralizumab efficacy in pediatric populations differs from that of other biologics and relies largely on extrapolation from adult trials and on pediatric pharmacokinetic and open-label studies (67).

FDA authorization of benralizumab for children 6–11 years was supported by the TATE study, a 48-week, open-label trial assessing pharmacokinetics and pharmacodynamics in 28 pediatric patients with severe eosinophilic asthma (mean age ≈ 9 years). The study reported an approximate 50% reduction in exacerbations and improved symptom control. Safety was favorable; the most common adverse events were mild upper-respiratory infections and injection-site reactions. Anti-drug antibodies occurred in 14.3% of participants without apparent impact on efficacy or safety. Additional pediatric trials are ongoing in the UK, enrolling children 6–18 years (42, 68).

Beyond maintenance therapy, there is growing interest in the potential role of benralizumab in the treatment of acute asthma exacerbations. In adults, the ABRA trial suggested that benralizumab administered during acute exacerbations may accelerate recovery and reduce subsequent exacerbation risk. However, no clinical trials have evaluated the efficacy or safety of this approach in pediatric asthma to date, and its use in children cannot currently be recommended outside of research settings (69).

Reslizumab, another anti–IL-5 monoclonal antibody, reduces eosinophilic inflammation and exacerbations in adults; however, its approval is limited to adults at present because pediatric evidence remains insufficient for routine use (57).

Following the established efficacy of mepolizumab, benralizumab, and reslizumab on the IL-5 axis, next-generation agents are being developed to extend durability and simplify dosing.

Depemokimab is an ultra–long-acting monoclonal antibody with enhanced affinity for IL-5 that enables 6-month dosing intervals (70). In two replicate, phase 3A, randomized, placebo-controlled trials (SWIFT-1 and SWIFT-2) enrolling adults with severe eosinophilic asthma (eosinophils ≥300/µL in the prior 12 months or ≥150/µL at screening; persistent exacerbations despite medium/high-dose ICS), patients received depemokimab 100 mg SC at weeks 0 and 26 or placebo, on top of standard care. Across trials (*n* = 762 full analysis set; 502 depemokimab; 260 placebo), depemokimab significantly reduced the annualized exacerbation rate vs. placebo (SWIFT-1: 0.46 [95% CI: 0.36–0.58] vs. 1.11 [0.86–1.43]; rate ratio 0.42, 95% CI: 0.30–0.59; *P* < 0.001; SWIFT-2: 0.56 [0.44–0.70] vs. 1.08 [0.83–1.41]; rate ratio 0.52, 95% CI: 0.36–0.73; *P* < 0.001). No between-group difference was observed for SGRQ change; due to hierarchical testing, no formal inference was drawn for subsequent secondary endpoints (FEV_1_, symptom scores) (70). Adverse-event rates were similar in both groups. Collectively, these findings support depemokimab as a twice-yearly anti-IL-5 option in severe eosinophilic asthma; pediatric studies are needed to define efficacy, safety, and positioning in children.

### 3.3 Anti-IL-4 Rα therapy (dupilumab)

The type-2 inflammatory response is orchestrated by CD4^+^ Th2 cells and type-2 innate lymphoid cells (ILC2s). IL-4 and IL-13 are pivotal mediators that drive Th2 differentiation, B-cell class switching to IgE, eosinophil recruitment, mucus hypersecretion, nitric oxide production by airway epithelium, bronchial hyperresponsiveness, and airway remodeling. Dupilumab is a fully human monoclonal antibody that binds the IL-4 receptor alpha (IL-4Rα) subunit, thereby blocking signaling of both IL-4 and IL-13. This dual inhibition downregulates T2-inflammatory pathways and mitigates hallmark features of T2 asthma; treatment is associated with reductions in biomarkers such as blood eosinophils and FeNO (71).

Dupilumab is approved as add-on maintenance therapy for children ≥6 years with moderate-to-severe asthma uncontrolled despite high-dose ICS plus at least one additional controller. In practice, biomarker-guided selection is recommended: blood eosinophils ≥150 cells/µL, FeNO ≥20 ppb, or both identify children with T2-high disease who are more likely to benefit from IL-4/IL-13 pathway blockade, consistent with GINA guidance (72).

In the phase III VOYAGE study, dupilumab significantly decreased the annual rate of severe asthma exacerbations in children with uncontrolled, moderate-to-severe asthma. Over the 52-week treatment period, 78% of children receiving dupilumab remained exacerbation-free, as compared with 60% in the placebo group, indicating a clinically meaningful reduction in risk. Improvements in lung function were rapid and durable: percent-predicted FEV_1_ (ppFEV_1_) was already significantly higher with dupilumab by week 2 and these gains were maintained through week 52. Benefit was observed across the type-2 (T2) phenotype and in biomarker-defined subgroups. In prespecified subgroup analyses, children with baseline blood eosinophils ≥300 cells/μL or FeNO ≥20 ppb experienced the most pronounced reductions in exacerbations and the greatest improvements in lung function, supporting a biomarker-guided approach to patient selection and consistent with dupilumab's mechanism of IL-4/IL-13 pathway blockade. Taken together, these findings endorse dupilumab as an effective add-on therapy for pediatric T2-high asthma, with early onset of action and sustained efficacy over one year (73).

A *post hoc* analysis comparing dupilumab plus medium-dose ICS with placebo plus continued high-dose ICS showed significant FeNO reductions with dupilumab throughout treatment. For blood eosinophils, the median change from baseline became negative by week 12 in the dupilumab group and remained lower through week 52 (–130 cells/µL with dupilumab vs. −70 cells/µL with placebo at week 52). Dupilumab plus medium-dose ICS also produced significant declines in serum total IgE over 52 weeks, unlike placebo plus high-dose ICS (74).

Clinically, adding dupilumab to medium-dose ICS—vs. persisting with high-dose ICS (with or without an additional controller)—led to a 74.3% reduction in severe exacerbations over 52 weeks, alongside greater improvements in lung function (ppFEV_1_) and asthma control. These results indicate that dupilumab can substantially reduce reliance on high-dose ICS in children with moderate-to-severe uncontrolled asthma, potentially lowering steroid-related risks while improving overall outcomes (74).

Across multiple studies, dupilumab has been associated with transient blood eosinophilia in approximately 4.1%–14% of treated patients vs. 0.6%–1% with placebo. The leading explanation is impaired tissue trafficking—dupilumab blocks IL-4/IL-13–mediated eosinophil migration without directly increasing bone-marrow production—yielding a temporary rise in circulating counts (42).

In a pre-specified analysis by Bacharier et al., both blood eosinophils and FeNO emerged as predictive and prognostic biomarkers of response. Children with eosinophils ≥150 cells/μL or FeNO ≥20 ppb had the greatest reductions in exacerbations and the largest lung-function gains, with maximal benefit in those meeting both criteria; conversely, outcomes were minimal when eosinophils <150 cells/μL and FeNO <20 ppb (75).

Long-term data from the EXCURSION open-label extension indicate that benefits persist for at least two years with continued use: children remaining on dupilumab maintained improved lung function and reduced exacerbation rates, while those switching from placebo experienced rapid clinical improvements. The safety profile was consistent with prior trials, with mild upper-respiratory infections and injection-site reactions most commonly reported and no new safety signals over extended follow-up (76).

Dupilumab delivers durable clinical benefit in pediatric T2-high asthma—particularly when eosinophils and/or FeNO are elevated—with a reassuring long-term safety profile. Its biomarker-guided use makes it a key biologic option in contemporary pediatric asthma management.

### 3.4 Anti-TSLP (tezepelumab) and other emerging agents

Recent advances have ushered in the so-called “epithelial era” of asthma research, in which the airway epithelium is recognized not merely as a barrier but as an active sentinel that shapes immune surveillance and inflammation (77).

In patients with asthma—especially those with severe disease—environmental exposures (allergens, viruses, pollutants, cleaning agents) can injure the epithelial barrier. The damaged epithelium then releases cytokines and growth factors that act on neighboring immune and structural cells, driving airway inflammation and structural remodeling, including subepithelial fibrosis, inflammatory angiogenesis, and thickening of the airway wall (76).

Among these epithelial-derived mediators, the alarmins—TSLP, IL-33, and IL-1α—play pivotal roles in initiating and sustaining type-2 immunity. Barrier dysfunction amplifies alarmin release, heightening pro-inflammatory activity and promoting a T2-skewed response (78).

This mechanistic insight has catalyzed development of next-generation biologics that target upstream drivers of inflammation, with the potential to benefit a broader spectrum of asthma phenotypes, including those less responsive to traditional T2-pathway inhibitors. Within this framework, tezepelumab (anti-TSLP) and other novel agents represent a new frontier for the management of severe pediatric asthma (41).

Tezepelumab is a fully human monoclonal antibody that neutralizes thymic stromal lymphopoietin (TSLP), an upstream epithelial alarmin that orchestrates type-2 immunity. By blocking TSLP, tezepelumab can blunt initiation of the inflammatory cascade, modulating not only downstream T2 pathways but also elements of non-T2 inflammation—supporting potential benefit in patients with low biomarker profiles or corticosteroid-refractory disease (79).

Tezepelumab is approved for adolescents ≥12 years with severe uncontrolled asthma despite high-dose ICS plus at least one controller, regardless of baseline eosinophil count. The recommended dose is 210 mg subcutaneously every 4 weeks (80).

Clinical efficacy has been demonstrated in adults and adolescents. In the phase III NAVIGATOR trial, tezepelumab reduced the annualized exacerbation rate by 56% overall, with reductions up to 71% among participants with blood eosinophils ≥300 cells/μL (81, 82). In prespecified subgroup analyses, a trend toward a reduction of approximately 30% in the annualized asthma exacerbation rate compared with placebo was observed in adolescents aged 12–18 years, although the trial was not powered to detect statistically significant differences within this age subgroup. Notably, benefit was also observed in groups that typically respond poorly to conventional T2 biologics—those with eosinophils <150 cells/μL and low FeNO. In the NAVIGATOR trial, the annualized asthma exacerbation rate was reduced by 39% among patients with baseline blood eosinophil counts <150 cells/μL and by 32% among those with FeNO <25 ppb, highlighting clinically meaningful benefit even in patients with lower levels of type-2 inflammatory biomarkers. Across analyses, improvements were seen in lung function, asthma control, and health-related quality of life (81, 82). However, the corticosteroid-sparing effect of tezepelumab remains uncertain. In the SOURCE trial, tezepelumab did not significantly reduce maintenance oral corticosteroid dose compared with placebo among patients with oral steroid-dependent severe asthma, underscoring the need for caution when extrapolating steroid-sparing expectations to pediatric practice (83).

In children aged 6 to <12 years, an ongoing phase III trial (NCT06023589) is evaluating the safety, efficacy, and pharmacokinetics of tezepelumab for severe, uncontrolled asthma; its results are expected to inform regulatory decisions and guide clinical use in younger pediatric populations (84).

Beyond tezepelumab, development is advancing for biologics that target epithelial cytokines and other upstream mediators. Anti–IL-33 monoclonal antibodies such as itepekimab and etokimab block the IL-33/ST2 axis, a key driver of ILC2 activation and eosinophilic inflammation; early adult studies have reported reductions in exacerbations and symptom burden (77). Tralokinumab, an IL-13–specific antibody with established benefit in atopic dermatitis, is being investigated for asthma phenotypes primarily driven by IL-13 (85). Additional programs are exploring inhibitors of the IL-1 family, TSLP receptor antagonists, and agents modulating IL-4/IL-13 and IL-17 pathways, reflecting the complex cytokine networks that shape T2 and non-T2 responses (77).

Collectively, these approaches represent the next wave of precision medicine in pediatric asthma. Their ultimate place in care will depend on outcomes from pediatric trials, validation of epithelial and immune biomarkers, and integration of molecular profiling to personalize treatment selection.

Approved biologics for severe pediatric asthma, including their molecular targets, approved age ranges, dosing regimens, and key biomarker signals, are summarized in Table 1.

**Table 1:** Approved biologics for severe pediatric asthma: targets, age, dosing, and biomarker signals.

Drug	Target	Age approval	Typical dosing	Biomarker signals for response	Key outcomes	Level of pediatric evidence
Omalizumab	IgE	≥6 years	SC q2–4w (weight/IgE-based)	Sensitization and total IgE primarily serve as eligibility criteria rather than predictive biomarkers; higher FeNO/eosinophils may be associated with greater benefit	↓ exacerbations, OCS-sparing not consistently demonstrated	High (multiple pediatric RCTs)
Mepolizumab	IL-5	≥6 years	40 mg (6–11 y), 100 mg (≥12 y) q4w	Higher blood eosinophils and T2-high phenotype (predictive signal supported, but pediatric-specific data remain limited)	↓ exacerbations; ↓ eosinophils; evidence for OCS-sparing in adults	High (pediatric RCTs available)
Benralizumab	IL-5Rα	≥6 years	30 mg (≥35 kg) or 10 mg (<35 kg): q4w × 3 then q8w	Higher blood eosinophils and T2-high phenotype	Near-complete eosinophil depletion; ↓ exacerbations; OCS-sparing demonstrated in adults	Low (no pediatric RCTs; PK/open-label only)
Dupilumab	IL-4Rα (IL-4/IL-13)	≥6 years	Label-based pediatric dosing q2w	Eosinophils ≥150/µL and/or FeNO ≥20 ppb (predictive signal across age groups)	↓ exacerbations; ↑ lung function (similar magnitude to other biologics in RCTs); OCS-sparing demonstrated in adults	High (pediatric RCTs available)
Tezepelumab	TSLP	≥12 years	210 mg q4w	Effective across biomarker strata, with greater effect in patients with higher T2 biomarker levels	↓ exacerbations incl. low-T2 groups; OCS-sparing not confirmed	Low-to-moderate (approved ≥12 y; pediatric-only RCTs lacking)

## 4 Discussion

Biologic therapy should be considered for severe asthma that remains uncontrolled despite optimized, maximal therapy and correction of modifiable factors (4, 31). Determining the endotype is essential before choosing a biologic, because each agent targets specific inflammatory pathways.

In practice, selection integrates regulatory age eligibility with biomarker-defined disease biology. As of GINA 2025, omalizumab, mepolizumab, and dupilumab are approved from ≥6 years, whereas benralizumab and tezepelumab are approved from ≥12 years (31).

Endotype assessment relies on key biomarkers. Omalizumab requires allergic sensitization (positive SPT or serum specific IgE) and total IgE within the dosing range (30–1,500 IU/mL) (3). Dupilumab is favored when blood eosinophils ≥150 cells/mm^3^, FeNO ≥20 ppb, or both are present, consistent with a T2-high profile (31). Anti–IL-5/IL-5R options, mepolizumab and benralizumab, are indicated for eosinophilic asthma, typically using ≥150 cells/µL at screening or ≥300 cells/µL in the prior year as practical thresholds for mepolizumab (31). Because tezepelumab targets TSLP upstream and modulates both T2 and non-T2 cascades, it may benefit children with lower biomarker levels or suboptimal corticosteroid responsiveness (80).

Comorbid indications can also steer choice. Per EMA labeling, omalizumab is approved for chronic spontaneous urticaria, mepolizumab for eosinophilic granulomatosis with polyangiitis, and dupilumab for atopic dermatitis and eosinophilic esophagitis—overlaps that may prioritize one biologic over another in pediatrics (86). Overall, a biomarker-guided, age-appropriate, and comorbidity-informed algorithm, applied after adherence/technique checks and treatable traits are addressed, remains the most rational strategy to personalize biologic therapy in severe pediatric asthma.

Optimizing adherence starts with clear education for the child and family on how the biologic is used, its safety profile, and the treatment goals. Better asthma control translates into tangible daily benefits, improved sleep, fewer school absences, and greater participation in sports and social activities (77).

When response is suboptimal, address practical barriers first: confirm adherence, injection technique/device handling, and interval timing; then evaluate coexisting factors (e.g., obesity and other comorbidities), psychosocial issues, and logistical constraints (family schedules, transport). Dosing frequency can influence fit and persistence: dupilumab every 2 weeks, omalizumab every 2–4 weeks, and mepolizumab and tezepelumab every 4 weeks (note that benralizumab is every 8 weeks after three 4-weekly loading doses) (29, 31, 80). Most biologics are administered subcutaneously, either at home or in clinic according to local labeling and practice; when feasible, home administration can mitigate logistical and psychological burdens and improve continuity.

Finally, shared decision-making, including discussion of dosing schedules, place of administration, anticipated benefits/risks, and use of reminders or teach-back for technique, enhances engagement and persistence, especially in adolescents who are transitioning to self-management.

Structured follow-up is essential to sustain adherence and to verify that treatment remains appropriate for the individual child. Assessment should integrate symptoms and asthma-specific quality of life (standardized questionnaires), lung function (e.g., spirometry with bronchodilator response where indicated), and periodic biomarker re-evaluation (blood eosinophils, FeNO, IgE where relevant). These data inform whether the current biologic (and dose interval) remains suitable or requires adjustment. When patients achieve and maintain good control, long-term continuation is reasonable; however, in pediatrics the criteria and optimal timing for dose reduction or withdrawal are not yet established and remain an area of active study (87).

Real-world evidence from the SPACE registry—one of the largest European cohorts of children with severe asthma receiving biologics (*n* = 250)—shows that most children attain good symptom control, yet ∼one third had partly controlled or uncontrolled asthma at the end of follow-up (88). Through its expert network, SPACE seeks to build consensus and provide guidance on continuation, switching, or discontinuation of biologic therapy to support informed, individualized decisions in practice.

Finally, biologics entail substantial costs, and access varies by country and insurance policy. Weighing price against expected benefits—fewer exacerbations and hospital visits, reduced steroid exposure, and improved quality of life—remains central to shared decision-making for families and clinicians and to payer approval processes (89, 90).

## 5 Conclusions

When children with severe asthma remain symptomatic despite optimized guideline-directed therapy, biologic treatment should be considered. Because these agents act on discrete inflammatory pathways, endotype determination is pivotal to selection and success. Choice of therapy should integrate the child's age/labeling, biomarkers (blood eosinophils, FeNO, IgE/sensitization), comorbidities (e.g., atopic dermatitis, EGPA, obesity), and psychosocial/family context, ensuring a regimen that is both biologically congruent and feasible in daily life.

Recent pediatric trials increasingly stratify by biomarkers and incorporate molecular endpoints, improving external validity; nevertheless, greater diversity in enrolled populations and broader coverage of real-world pediatric phenotypes are still needed. A standardized definition of pediatric asthma remission is lacking, and prospective evidence that biologics induce true remission in children is not yet available. Even so, framing remission (symptom freedom, exacerbation prevention, normalized lung function, minimal corticosteroid exposure, and biomarker quiescence) as a treatment goal could encourage earlier, more precise use of biologics and may ultimately support disease modification.

Key unresolved questions include the optimal duration of biologic therapy, criteria for tapering or discontinuation, and long-term immunologic safety in growing children. Importantly, pediatric asthma follows highly variable and evolving disease trajectories, ranging from remission to persistent or relapsing phenotypes. This heterogeneity makes it particularly difficult to define the optimal timing for initiating biologic therapy and the appropriate duration of treatment in children, reinforcing the need for longitudinal data and structured de-escalation strategies. Addressing these gaps—alongside equitable access and continued biomarker refinement—will be essential to realize the full promise of precision medicine in severe pediatric asthma (87, 88).

Access to biologic therapies remains uneven globally owing to variation in regulatory approvals, reimbursement policies, and health-system capacity. Expanding equitable access to precision medicine is a priority, particularly in low-resource settings.

Pediatricians, allergists, and pulmonologists should be fluent in indications, mechanisms, and practical implementation of biologics—including eligibility criteria, dosing logistics, reimbursement pathways, and monitoring requirements. Strengthened multidisciplinary models—linking specialty care with clinical pharmacology, behavioral support, and school-based interventions—can maximize real-world benefit.

Clinical guidelines should evolve to incorporate transparent biologic selection tools, structured step-up/step-down algorithms, and shared decision-making frameworks tailored to families. Future research must establish standard definitions of treatment success (including remission) and provide clear criteria for dose reduction or discontinuation, with long-term pediatric outcomes and safety explicitly addressed.
